# The Roles of SQSTM1/p62 in Selective Autophagy and Oncogenic Signaling

**DOI:** 10.3390/ijms27052342

**Published:** 2026-03-02

**Authors:** Young-Jun Kim, Hwa-Hyeong Lee, Tae Young Jung, Young-Hoon Jeong, Key-Hwan Lim, Ji Min Han

**Affiliations:** College of Pharmacy, Chungbuk National University, Osongsaengmyeong 1-ro, Osong-eup, Heungdeok-gu, Cheongju-si 28160, Republic of Korea; youngjun9962@gmail.com (Y.-J.K.);

**Keywords:** autophagy, cancer, oncogene, p62, ubiquitin

## Abstract

Autophagy is a critical cellular mechanism that regulates the degradation of misfolded and aggregated proteins and non-functional intracellular organelles. Based on the fundamental qualities of the substrates targeted for degradation and the distinct molecular mechanisms involved, autophagy can be classified into three major types: macroautophagy, microautophagy, and chaperone-mediated autophagy (CMA). Sequestosome 1 (SQSTM1)/p62, which functions as a signaling hub integrating nuclear factor kappa B (NF-κB), the mechanistic target of rapamycin complex 1 (mTORC1), and Kelch-like ECH-associated protein 1 (Keap1)–nuclear factor erythroid 2–related factor 2 (NRF2) pathways, serves as a selective macroautophagy/autophagy receptor that binds ubiquitinated cargo proteins and recruits them to the autophagosome for subsequent degradation in the autolysosome. Furthermore, the phase separation of p62 is an important regulatory process in the autophagy mechanism, but recent studies have demonstrated that impaired or excessive autophagy mediated by p62 is associated with cancer development. This review summarizes the role of autophagy—including its types, mechanisms, and the pathway related to the ubiquitin-dependent selective autophagy receptor p62—in cancer progression.

## 1. Introduction

In eukaryotic cells, two major degradative pathways maintain protein homeostasis: the ubiquitin–proteasome system (UPS) and autophagy [1]. The UPS primarily targets small misfolded or damaged proteins for degradation, whereas autophagy is responsible for degrading larger protein aggregates and damaged organelles [2]. Briefly, the UPS is initiated by the post-translational modification (PTM) of proteins through ubiquitination, which serves as a signal for their recognition and subsequent degradation by the proteasome [3].

Christian de Duve, who discovered lysosomes, provided the first piece of evidence for their involvement in the autophagy mechanism, leading to the introduction of the term “autophagy” [4]. Subsequently, autophagy was examined in the liver of animals under nutrient-deprivation conditions, although it was not a primary area of inquiry at the time [5]. In the 1990s, autophagy-related genes (ATGs) were first identified in yeast, emphasizing the lasting significance of autophagy research [6]. These ATGs mediate autophagosome formation, and to date, more than 40 ATGs have been identified, including the core genes of autophagy-related gene 1 (ATG1) through autophagy-related gene 18 (ATG18) [7]. Autophagy is a catabolic process that provides an alternative energy source [8] and is induced in response to various conditions, such as cellular stress and starvation [9]. Based on substrate specificity, autophagy is categorized into non-selective and selective types. This process serves as a crucial regulator of cellular homeostasis, and autophagy dysfunction has been associated with numerous diseases, including aging, metabolic disorders, cancer, inflammation, infections, and neurodegenerative diseases [7]. The selective autophagy adaptor Sequestosome 1 (SQSTM1)/p62 functions as a multifunctional signaling hub that interacts with signaling proteins through distinct functional domains [10]. Additionally, p62 functions as a ubiquitin sensor and regulates the nuclear factor kappa B (NF-κB) pathway through the aggregation and ubiquitination of tumor necrosis factor receptor-associated factor 6 (TRAF6) [11]. In the context of autophagy, the ubiquitin-associated (UBA) domain of p62 binds to ubiquitinated cargo, whereas the microtubule-associated protein 1 light chain 3 (LC3)-interacting region (LIR) domain of p62 binds to LC3, thereby directly recruiting ubiquitinated cargo to the autophagic machinery [12]. Consequently, autophagy deficiency leads to p62 aggregation, which contributes to the development of various diseases [13]. Furthermore, the multiple domains of p62 are involved in diverse signaling pathways that promote carcinogenesis [14]. Moreover, PTMs of p62 play a crucial role in regulating both p62 phase separation and autophagic activity [15]. Therefore, in this review, we provide an overview of the molecular mechanisms underlying autophagy, followed by an in-depth discussion of selective autophagy—particularly concerning the role of p62—and its significance in cancer.

## 2. Role of Autophagy in Cellular Homeostasis

**Figure 1:**
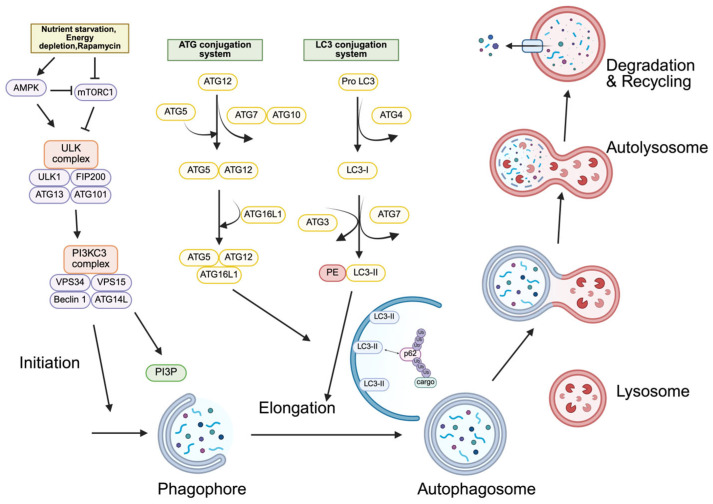
The mechanism of autophagy in cells. Stressful conditions, including nutrient deprivation and energy depletion, upregulate AMP-activated protein kinase (AMPK) and inhibit the activity of mechanistic target of rapamycin complex 1 (mTORC1). This leads to activation of the Unc-51–like kinase 1 (ULK1) complex, which consists of autophagy-related gene 13 (ATG13), autophagy-related gene 101 (ATG101), FAK family kinase–interacting protein of 200 kDa (FIP200), and ULK1. The Unc-51–like kinase (ULK) complex then activates the class III phosphatidylinositol 3-kinase (PI3KC3) complex, composed of vacuolar protein sorting 34 (VPS34), vacuolar protein sorting 15 (VPS15), Beclin-1, and autophagy-related gene 14-like (ATG14L). The PI3KC3 complex produces phosphatidylinositol 3-phosphate (PI3P), which induces the initiation of phagophore formation. Autophagosome formation proceeds in two steps: the autophagy-related gene (ATG) conjugation system and microtubule-associated protein 1 light chain 3 (LC3) conjugation system. The autophagy-related gene 5 (ATG5)–autophagy-related gene 12 (ATG12)–autophagy-related gene 16-like 1 (ATG16L1) complex and microtubule-associated protein 1A/1B-light chain 3-II (LC3-II) promote elongation of the autophagosome. Autophagy receptor p62 binds to ubiquitinated cargo, then forming autolysosome. The autolysosome degrades all the substrates in the autophagosome.

**Figure 2:**
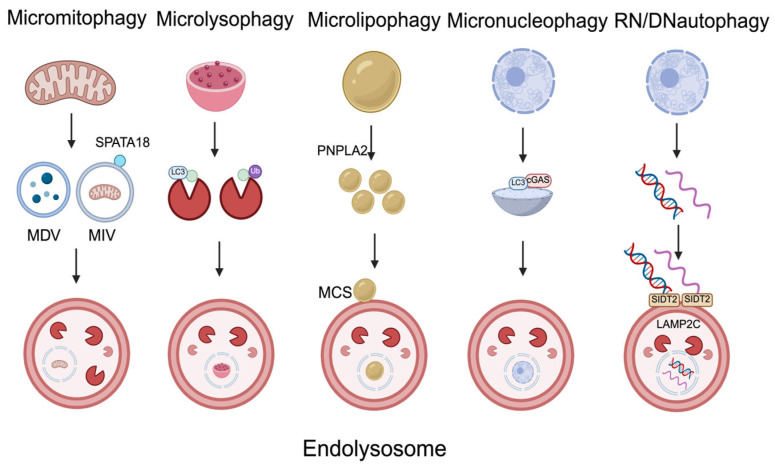
The mechanism of selective microautophagy. The mechanism of micromitophagy involves endolysosomal degradation of mitochondria through the formation of mitochondrial-derived vesicles (MDVs), whereas another mechanism involves the formation of a spermatogenesis-associated protein 18 (SPATA18)-induced vacuole (MIV), which mediates endolysosomal degradation of damaged mitochondria. The mechanism of microlysophagy involves the degradation of damaged lysosome-related proteins through an LC3-dependent pathway mediated by the formation of intralumenal vesicles and a ubiquitination-dependent pathway. The mechanism of microlipophagy involves the lipolysis of large lipid droplets mediated by adipose triglyceride lipase (PNPLA2). The resulting smaller lipid droplets form stable contacts with lysosomes, known as membrane contact sites (MCSs), through which they are degraded. The mechanism of micronucleophagy involves the engulfment of micronuclei by endolysosomes through LC3 binding, which is mediated by cyclic GMP–AMP synthase (cGAS). The mechanism of RNA/DNA autophagy involves the degradation of RNA and DNA through direct transport to lysosomes, which is mediated by lysosomal-associated membrane protein 2C (LAMP2C) and SID1 transmembrane family member 2 (SIDT2).

**Figure 3:**
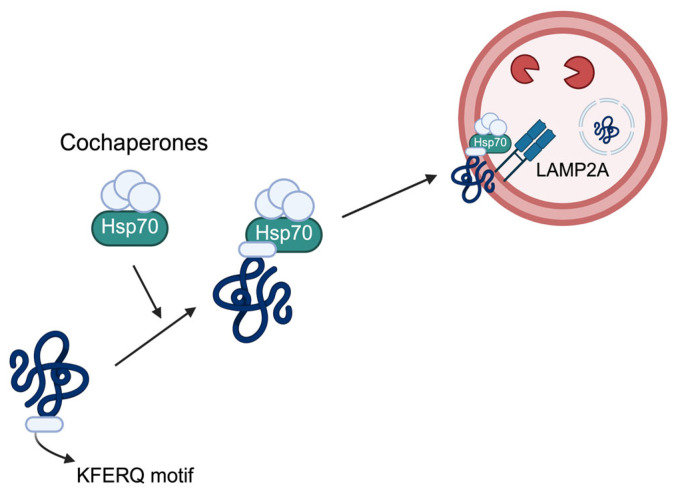
The mechanism of chaperone-mediated autophagy (CMA) involves the degradation of targeted proteins containing KFERQ-like motifs. Cochaperones, such as heat shock cognate 70 (HSC70), the HSP70-interacting protein (CHIP), heat shock protein 40 (HSP40), and HSP70–HSP90 organizing protein (HOP), mediate their translocation to lysosomes, in which they are recognized by the receptor lysosome-associated membrane protein type 2A (LAMP2A), resulting in selective degradation within lysosomes.

Autophagy is a critical cellular mechanism that regulates the degradation of misfolded proteins, non-functional intracellular organelles, lipids, nucleic acids, and aggregated proteins [7]. This process is an evolutionarily conserved catabolic mechanism [5] that is essential for maintaining cellular homeostasis [7]. Macroautophagy (Figure 1), microautophagy (Figure 2), and chaperone-mediated autophagy (CMA) (Figure 3) constitute the three major categories of autophagy, which are classified according to the substrates targeted for degradation and the distinct molecular mechanisms involved [16]. Furthermore, autophagy can be divided into two main types—selective autophagy and non-selective (or bulk) autophagy—based on substrate specificity. Non-selective autophagy refers to the non-specific engulfment and degradation of cytoplasmic components, such as macromolecular complexes and organelles, while selective autophagy specifically targets certain substrates for degradation. For instance, mitophagy targets mitochondria, lysophagy targets lysosomes, aggrephagy targets protein and RNA aggregates, endoplasmic reticulum (ER)-phagy targets the ER, pexophagy targets peroxisomes, ribophagy targets ribosomes, ferritinophagy targets ferritin, glycophagy targets glycogen, lipophagy targets lipid droplets, and fluidophagy targets fluid-filled vesicles [7]. Among these forms, selective autophagy is particularly relevant to cancer biology, as cargo receptors such as p62 mediate substrate recognition and link autophagic degradation pathways to cellular stress signaling networks.

### 2.1. Autophagy Types

#### 2.1.1. Macroautophagy

Macroautophagy, the most extensively studied form of autophagy, involves the formation of a double-membrane structure known as the phagophore, which elongates and closes to generate the autophagosome, enclosing cytoplasmic components destined for degradation. Subsequently, the autophagosome fuses with the lysosome, resulting in the formation of the autolysosome, where the enclosed materials are degraded by lysosomal enzymes [17]. Autophagy initiation is triggered by nutrient starvation, energy depletion, and cellular stress [16]. Under these conditions, the mechanistic target of rapamycin complex 1 (mTORC1) becomes inactivated, leading to the activation of the Unc-51-like kinase (ULK) complex near the ER. The ULK complex comprises Unc-51-like kinase 1 (ULK1), ATG101, ATG13, and the FAK family kinase–interacting protein of 200 kDa (FIP200) (also known as RB1CC1) [18]. Furthermore, the activation of ULK1 is regulated by the enzymatic activity of AMP-activated protein kinase (AMPK) [19]. The autophagy adaptor p62 contains an LIR domain that directly interacts with the C-terminal region of FIP200, thereby promoting the recruitment of the ULK complex [20]. The class III phosphatidylinositol 3-kinase complex I (PI3KC3–C1) consists of lipid kinase vacuolar protein sorting 15 (VPS15), vacuolar protein sorting 34 (VPS34), the tumor suppressor Beclin-1, and the autophagy-specific subunit ATG14 [21], which is recruited by the ULK complex [18]. Upon binding to the autophagic membrane, PI3KC3–C1 generates phosphatidylinositol 3-phosphate [PI(3)P] on the membrane surface [22]. The elongation of the autophagosome is regulated through a complex mechanism involving ubiquitin-like conjugation systems, particularly the ATG8 and ATG12 conjugation pathways [18]. The ATG12–ATG5 conjugate associates with ATG16L to form the ATG12–ATG5–ATG16L complex [23]. Similarly, the ubiquitin-like protein ATG8 is conjugated to the E2-like enzyme ATG3 through the E1-like enzyme ATG7 [24], followed by the conjugation of phosphatidylethanolamine (PE) to ATG8. Members of the ATG8 family (known as LC3 proteins in mammals) play a crucial role in autophagosome elongation through the E3-like activity of the ATG12–ATG5–ATG16L1 complex [18]. The conversion of LC3-I to LC3-II is mediated by the covalent attachment of LC3-I to PE [25]. In selective autophagy, the LIR motif of autophagy adaptors, which also contains a ubiquitin-binding domain, interacts with LC3 [7]. Subsequently, the autophagosome fuses with the lysosome to form the autolysosome [26], where selectively ubiquitinated cargo is degraded, and autophagy adaptors that have completed their function are themselves degraded within the lysosome.

#### 2.1.2. Microautophagy

Microautophagy, which has been studied far less extensively than macroautophagy and CMA, is characterized by the direct sequestration of cytoplasmic components into lysosomes or late endosomes without the formation of autophagosomes [27]. The sequestered substrates are subsequently degraded within the endolysosomal lumen [5], and the transport of autophagic cargo to lysosomes and late endosomes occurs through diverse processes, including lysosomal protrusion, lysosomal invagination, and endosomal invagination [28]. Recent studies on non-selective microautophagy have revealed that this process can be classified into two types: (1) fission-type microautophagy, mediated by endosomal sorting complexes required for transport machinery, and (2) fusion-type microautophagy, mediated by the soluble N-ethylmaleimide-sensitive factor attachment protein receptor complex and the core autophagy machinery [5]. Moreover, increasing attention has been directed toward selective forms of microautophagy, and similarly to macroautophagy, each type is defined by its specific target cargo. The mechanism of micromitophagy involves the formation of spermatogenesis-associated protein 18 (SPATA18)-induced vacuoles [5] and mitochondrial-derived vesicles (MDVs) [29]. Microlysophagy proceeds via an LC3-dependent pathway, which involves the formation of intraluminal vesicles [30], and a ubiquitination-dependent pathway [31]. In microlipophagy, adipose triglyceride lipase (PNPLA2) hydrolyzes large lipid droplets into smaller ones that are subsequently delivered to lysosomes for degradation [32]. Micronucleophagy is mediated by cyclic GMP–AMP synthase (cGAS), which promotes the engulfment of micronuclei [33]. RNA/DNA autophagy is facilitated by lysosomal-associated membrane protein 2C (LAMP2C) and SID1 transmembrane family member 2 (SIDT2), which mediate the transport of RNA and DNA into lysosomes [34]. However, compared to macroautophagy, the mechanistic contribution of microautophagy to p62-dependent signaling remains less clearly defined.

#### 2.1.3. Chaperone-Mediated Autophagy (CMA)

CMA, first identified as a mechanism for the selective degradation of autophagic cargo [35], specifically targets proteins containing a KFERQ-like motif [36]. The targeted proteins interact with the 70 kDa heat shock cognate protein (HSC70) [37], and at the lysosomal membrane, lysosome-associated membrane protein type 2A (LAMP-2A) recognizes target proteins bound to HSC70 [38]. Upon binding, LAMP-2A undergoes multimerization to form a translocation complex. The substrate protein is then translocated into the lysosomal lumen with the assistance of luminal chaperones, leading to its degradation [39]. Although CMA functions independently of p62, the coordinated regulation among different autophagic pathways may collectively modulate cellular proteostasis and stress signaling in cancer.

## 3. Selective Autophagy Receptor SQSTM1/p62

**Figure 4:**
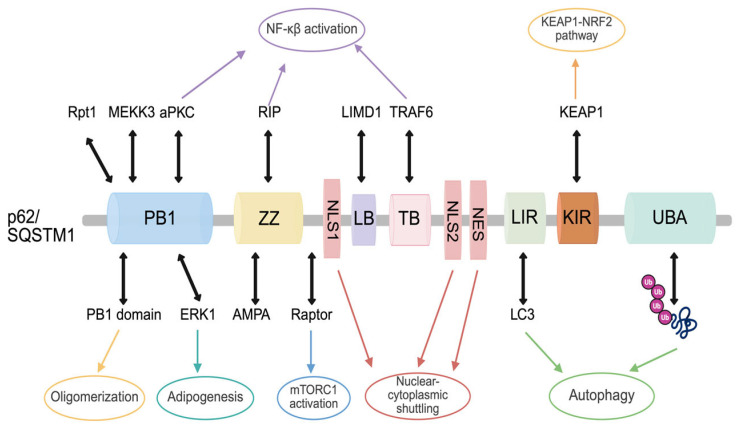
The p62 domain structure, interaction partners, and functions. The Phox1 and Bem1p (PB1) domain forms oligomers through interactions with the PB1 domains of other proteins and interacts with regulatory particle triple-A ATPase 1 (Rpt1), mitogen-activated protein kinase kinase kinase 3 (MEKK3), atypical protein kinase C (aPKC), and extracellular signal-regulated kinase 1 (ERK1). The interaction with ERK1 regulates adipogenesis. The zinc finger (ZZ) domain interacts with the α-amino-3-hydroxy-5-methyl-4-isoxazolepropionic acid receptor (AMPA) receptor and receptor-interacting protein (RIP). The tumor necrosis factor receptor–associated factor 6 (TRAF6) binding domain (TB) binds to TRAF6. RIP, TRAF6, and aPKC are associated with nuclear factor kappa B (NF-κB) activation. The two nuclear localization signals (NLS1/2) and a nuclear export signal (NES) domain regulate nuclear-cytoplasmic shuttling. The autophagy regulation of p62 is associated with the LC3-interacting region (LIR) domain and the ubiquitin-associated (UBA) domain, which recognize ubiquitinated proteins and facilitate their sequestration into autophagosomes. Kelch-like ECH-associated protein 1 (Keap1) binds to the Keap1-interacting region (KIR) domain of p62, leading to disruption of the Keap1–nuclear factor erythroid 2–related factor 2 (NRF2) complex, thereby regulating NRF2 activity.

Among the autophagy adaptors with related functions, such as neighbor of BRCA1 gene 1 (NBR1), Tax1 binding protein 1 (TAX1BP1), nuclear dot protein 52 (NDP52), and optineurin (OPTN), p62 acts as a molecular scaffold and serves as a selective autophagy receptor [10] by binding to ubiquitinated cargo proteins targeted for degradation through autophagy [40]. p62 also contains multiple domains that regulate its interactions with various signaling proteins, thereby facilitating its role as a signaling hub (Figure 4).

### 3.1. SQSTM1/p62 Domains Related to Signaling Pathways

p62 possesses multiple functional domains, including a Phox and Bem1p (PB1) domain at the N-terminus, a Keap1-interacting region (KIR), an LIR, a zinc finger (ZZ) domain, a TRAF6-binding domain, a UBA domain, a nuclear export signal (NES) domain, and two nuclear localization signals (NLS1/2) domains [40]. Additionally, recent studies have identified the LIM protein-binding (LB) domain as another functional domain of p62 [14].

The PB1 domain of p62 forms heterodimers with other PB1-containing proteins, thereby regulating various signaling pathways and cellular processes [41]. Moreover, the PB1 domain interacts with atypical protein kinase C (aPKC) isoforms, which mediate cell proliferation and survival, regulate growth factor receptor trafficking, and control NF-κB activation [42]. The mitogen-activated protein kinase (MAPK) signaling components, namely mitogen-activated protein kinase kinase kinase 2 (MEKK2) and MEKK3, also interact with the PB1 domain of p62 [43], while the TRAF6-dependent binding of MEKK3 contributes to NF-κB activation [44]. Additionally, extracellular signal-regulated kinase 1 (ERK1) interacts with the PB1 domain of p62 to promote adipogenesis [45]. Furthermore, p62 binds to the 19S proteasomal subunits—regulatory particle triple-A ATPase 1 (Rpt1) and regulatory particle non-ATPase 10 (Rpn10)—via its PB1 domain, thereby facilitating the recruitment of ubiquitinated proteins to the proteasome [46].

The ZZ domain of p62 selectively interacts with receptor-interacting protein (RIP), thereby linking RIP to aPKCs, which contributes to the activation of the NF-κB signaling pathway [47]. Furthermore, the ZZ-type zinc finger domain of p62 interacts with the intracellular loop L2–3 of the glutamate receptor 1 (GluR1) subunit of the α-amino-3-hydroxy-5-methyl-4-isoxazolepropionic acid (AMPA) receptor, which is phosphorylated by aPKC [48]. Recent studies have demonstrated that the p62 ZZ domain binds to type-1 and type-2 N-terminal degrons (N-degrons) and interacts with N-terminal arginine, thereby functioning as an N-recognin and promoting p62-dependent autophagic activity as an autophagy adaptor [49].

The TRAF6-binding domain of p62 interacts with TRAF6, as its name suggests [40]. The p62–TRAF6 interaction activates the NF-κB signaling pathway, which influences tumor angiogenesis and invasion and is also linked to cancer-associated inflammation [50]. The oncogene Ras is upregulated by p62 through the mediation of NF-κB signaling [51]. The p62-TRAF6 complex regulates mTORC1 activation via TRAF6-catalyzed K63-linked ubiquitination of mTOR in amino acid-stimulated cells and facilitates the translocation of mTORC1 to the lysosome. This complex modulates autophagy and plays a crucial role in cancer cell proliferation [52]. Furthermore, the interaction between TRAF6 and p62 is essential for receptor activator of NF-κB-induced osteoclastogenesis [53].

p62 binds to ubiquitinated cargo proteins through its UBA domain, thereby functioning as a selective autophagy receptor that mediates cargo degradation [54]. Not only does phosphorylation of serine 409 in the UBA domain by the autophagy-related kinase ULK1 promote the interaction between p62 and ubiquitin and destabilize the UBA dimer interface [55], but phosphorylation of serine 403 in the UBA domain by casein kinase 2 (CK2) also further enhances the binding affinity of p62 to polyubiquitin chains [56].

LC3, a protein essential for autophagosome formation, interacts with the LIR domain of p62 [40], which is crucial for selective autophagy, as it mediates the specific recognition of ubiquitinated proteins destined for autophagosome-dependent degradation [14].

The Keap1–NRF2 signaling pathway is activated by phosphorylation of p62 during autophagy. The KIR domain of p62 directly binds to Keap1 through the KIR motif [57]. Under oxidative stress, upregulation of p62 is induced by the transcription factor NRF2, while p62, in turn, activates NRF2. The KIR motif, located adjacent to the LIR motif, shares structural similarity with the ETGE motif [58]. Moreover, p62-mediated autophagic degradation activates the NRF2 signaling pathway via Toll-like receptor-dependent signaling [59], while the LB domain of p62 binds to the prefoldin-like chaperone UXT, enhancing autophagic flux through the formation of p62 bodies [60]. The E3 ubiquitin ligase RNF168 promotes histone H2A ubiquitination and induces the formation of polyubiquitin chains under conditions of DNA damage. RNF168 interacts with the LB domain of p62, and this interaction inhibits RNF168 activity [61].

Although p62 is primarily a cytosolic protein, it contains two nuclear localization signals (NLS) domains and one NES domain that enable its shuttling between the nucleus and cytoplasm [62]. Phosphorylation near the NLS2 domain regulates the nucleocytoplasmic transport of p62 [62].

### 3.2. Post-Translational Modifications (PTMs) Regulating Cellular Functions of Selective Autophagy Receptor SQSTM1/p62

The phase separation of p62—which is regulated through the interaction of its UBA domain with ubiquitinated cargo—represents a crucial molecular mechanism in autophagy [63]. Moreover, this interaction is precisely modulated by PTMs of p62, including ubiquitination, acetylation, and phosphorylation [64]. In prostate cancer, speckle-type BTB/POZ protein (SPOP) mutations have been identified along with various other genetic alterations [65]. The non-degradative ubiquitination of p62 at lysine 420 (K420) within the UBA domain is mediated by the E3 ubiquitin ligase substrate-binding adaptor SPOP, resulting in the inhibition of p62-dependent autophagy [66]. The dimerization of the UBA domain of p62 reduces its binding affinity for ubiquitin [54]. In addition, multiple E3 ubiquitin ligases regulate p62-mediated signaling pathways through the ubiquitination of distinct p62 domains. For example, ubiquitination of p62 at K7 within the PB1 domain is mediated by the RING finger domain-containing E3 ubiquitin ligase TRIM21, leading to the inhibition of p62 oligomerization [67], and by the HECT-type E3 ubiquitin ligase NEDD4, which regulates p62-dependent inclusion body autophagy [68]. The E3 ligase Parkin, which is associated with Parkinson’s disease (PD), modulates the p62–Parkin axis by promoting the proteasomal degradation of p62 through ubiquitination at K13 in the PB1 domain [69]. The E3 ligase RNF166 mediates K29/K33-linked polyubiquitination of p62 at K91 in the PB1 domain and K189 in the linker region, resulting in the activation of xenophagy [70]. Recent studies have reported that aberrant ubiquitination of p62 at K281 within the linker region by the mutant SCF^cyclinF^ E3 ligase complex is associated with amyotrophic lateral sclerosis (ALS) and frontotemporal dementia [71]. Moreover, the E3 ligase TRIM 25 also interacts with p62 and mediates K63-linked ubiquitination, leading to the disruption of p62 oligomer formation and the consequent inhibition of autophagy activity [72]. Conversely, deubiquitinating enzymes (DUBs) regulate the ubiquitination status of p62. For instance, ubiquitin-specific protease 13 (USP13) removes the ubiquitin chain from p62 at K7 in the PB1 domain, promoting p62 oligomerization and consequently activating autophagy and inducing Keap1 degradation [73]. Moreover, OTU deubiquitinase 7B (OTUD7B) also removes ubiquitin from p62, leading to degradation of interferon regulatory factor 3 by stabilizing p62 [74]. Deubiquitination of p62 at K420 within the UBA domain by ubiquitin-specific protease 8 (USP8) inhibits its autophagic activity [75], and ubiquitin-specific protease 15 (USP15) negatively regulates RNF26-mediated ubiquitination of p62 [76].

The acetylation of p62 at K420 and K435 within its UBA domain is mediated by the acetyltransferase TIP60, resulting in an increased binding affinity between the UBA domain and ubiquitin [77]. In addition, deacetylation of p62 at K295 by SIRT1 antagonizes GCN5-mediated acetylation and upregulates p62 expression, thereby promoting hepatocellular carcinoma progression [78]. Furthermore, deacetylation of p62 at K264 by SIRT7 counteracts hMOF-mediated acetylation and facilitates DNA damage repair through interaction with apurinic/apyrimidinic endonuclease 1 (APE1) [79]. Recent studies have revealed a significant association between S-acylation and p62 [80] [81]. S-acylation is a PTM that covalently attaches fatty acids to cysteine residues of target proteins via a thioester linkage, a process catalyzed by S-acyltransferases [82]. The S-acylation of p62 at Cys289 and Cys290 is mediated by S-acyltransferase 19 (ZDHHC19), leading to enhanced membrane affinity of p62 droplets by increasing local hydrophobicity. Conversely, acyl-protein thioesterase 1 (LYPLA1/APT1) catalyzes the deacylation of p62 [80,81].

The phosphorylation of p62 at Ser403 within its UBA domain is mediated by ULK1 [83]. and is also promoted by transforming growth factor β-activated kinase 1 (TAK1) [84], TANK-binding kinase 1 (TBK1) [85], and CK2 [56], thereby increasing the binding affinity between the UBA domain and ubiquitin [64]. Furthermore, phosphorylation of p62 at Ser349 within its KIR domain is mediated by mTORC1 [86], protein kinase C delta (PKCδ) [87], PKR-like endoplasmic reticulum kinase (PERK) [88], and leucine-rich repeat kinase 2 (LRRK2) [89], thereby contributing to the regulation of the p62 signaling pathway. Recent studies have reported that phosphorylation of p62 at Ser207 and Thr269 is mediated by dual-specificity tyrosine-phosphorylation-regulated kinase (DYRK). In particular, phosphorylation of p62 at Thr269 enhances the interaction between p62 and TRAF6, leading to activation of mTORC1 and promoting melanoma progression [90]. In addition, phosphorylation of p62 at Thr269/Ser272 is mediated by cyclin-dependent kinase-like 5 (CDKL5), leading to the regulation of autophagy [91].

## 4. Association of Selective Autophagy Receptor SQSTM1/p62 with Cancers

**Figure 5:**
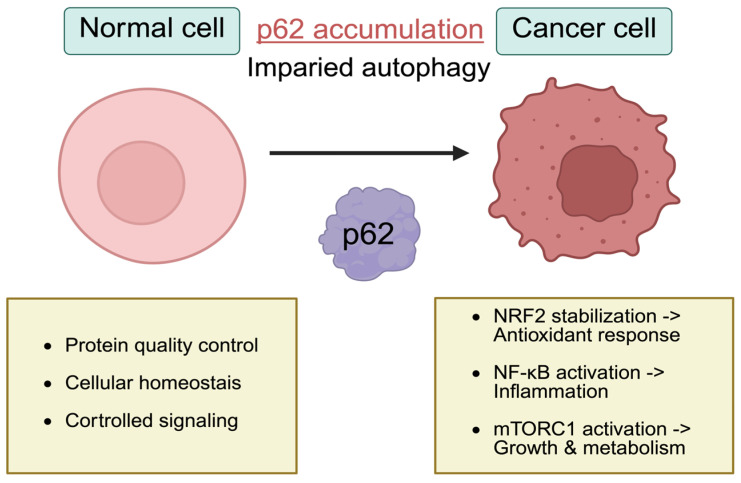
In normal cells, p62 functions as a selective autophagy receptor and signaling hub, contributing to protein quality control, maintenance of cellular homeostasis, and regulated signaling. In contrast, impaired autophagy in cancer cells leads to p62 accumulation, which promotes oncogenic signaling pathways. Aberrant p62 levels stabilize NRF2 to increase antioxidant response, activate NF-κB signaling to promote inflammation, and stimulate mTORC1 signaling to regulate tumor growth and metabolic reprogramming.

The selective autophagy receptor p62 serves as a regulatory integrator, but this multifunctionality can also confer oncogenic potential (Figure 5) [10]. Recent studies have demonstrated that p62 positively regulates the NF-κB signaling pathway, thereby contributing to cancer development and inflammatory responses [92], and aberrant p62 expression has been identified in various human cancers, suggesting its critical role in tumorigenesis and cancer progression (Table 1).

Rather than exerting consistent effects across tumor types, the oncogenic functions of p62 appear to be mediated through recurrent signaling modules that operate across distinct tissue contexts. These include the p62–KEAP1–NRF2 axis, the mTORC1-dependent metabolic axis, and alterations associated with impaired autophagic flux.

Among these mechanisms, the p62–KEAP1–NRF2 axis represents one of the most extensively characterized pathways linking selective autophagy to tumor adaptation. In hepatocellular carcinoma (HCC) cells, increased accumulation of p62 promotes the stabilization of NRF2 through direct interaction with p62, thereby activating the transcription of NRF2 target genes, and persistent activation of NRF2 contributes to the development of HCC [93]. Furthermore, phosphorylated p62 accumulation also promotes NRF2 activation, resulting in the growth of human HCCs [57]. In particular, phosphorylation of p62 at serine 349 (Ser349) confers resistance to anticancer drugs and enhances the proliferative capacity of HCC cells, leading to its accumulation in tumor regions positive for hepatitis C virus (HCV). Therefore, NRF2 inhibitors may be effective therapeutic agents for HCV-positive HCC patients [94]. Immunohistochemical analysis has shown that the combination of aminoacylase 1 (ACY1) + SQSTM1 + glypican 3 (GPC3) serves as a critical biomarker for distinguishing well-differentiated HCC from high-grade dysplastic nodules [95]. In human HCC tissues, autophagy is more defective than in surrounding non-tumorous liver tissues, and immunostaining revealed that both p62 and GPC3 expression are elevated [96].

Similar redox-adaptive mechanisms have been observed in other malignancies. In lung cancer, clinicopathologic analyses have shown that the accumulation of p62 and NRF2 is associated with reduced lung cancer-specific survival, while NRF2 status influences the prognosis of non-small cell lung cancer (NSCLC) regardless of histological subtype, whereas the prognostic significance of p62 is predominantly observed in adenocarcinoma [97]. Furthermore, in stage I/II NSCLC, LC3 expression is positively correlated with p62 expression. High p62 expression is associated with more aggressive tumors, whereas high LC3 expression is linked to less aggressive tumors [98]. In clear cell renal cell carcinoma (ccRCC), the most common subtype of kidney cancer, patients tend to gain chromosome 5q, whose amplification drives p62 overexpression in ccRCC cell lines, thereby conferring resistance to redox stress [99]. In oral carcinoma cells, p62 knockdown leads to ROS accumulation and glutathione (GSH) reduction, but it does not affect the Keap1-NRF2 pathway. Consequently, in oral epithelial carcinogenesis, p62 overexpression is significantly associated with GSH induction, which confers resistance to cytotoxic stresses such as chemotherapy and radiation [100]; however, this effect may occur independently of canonical KEAP1–NRF2 regulation. Importantly, despite differences in tissue context, p62-driven NRF2 activation consistently promotes redox adaptation in HCC, lung cancer, and renal malignancies, suggesting a conserved stress-adaptive mechanism across metabolically active tumors. Collectively, these findings indicate that p62-mediated NRF2 activation provides a selective survival advantage under oxidative and metabolic stress conditions common to rapidly proliferating tumors.

In addition to redox regulation, p62 intersects with metabolic signaling pathways. In HCC, hepatocyte-specific overexpression of p62 induces activation of NRF2, c-Myc, and mTORC1, thereby promoting the survival and expansion of ROS-containing HCC-initiating cells [101]. In thyroid cancer, papillary thyroid carcinoma (PTC) is the most prevalent histological subtype of thyroid cancer, and patients with PTC have overexpressed p62 in tumor tissues compared with normal thyroid tissues. Consistently, in PTC cell lines, p62 expression is higher than that in normal thyroid cell lines, and at the molecular level, it regulates autophagy and apoptosis in TPC-1 cells through the AMP-activated protein kinase (AMPK)/AKT/mTOR signaling pathway [102]. In prostate adenocarcinoma (PRAD), cytosolic p62 expression is elevated in prostatic adenocarcinoma and high-grade prostatic intraepithelial neoplasia (PIN) [103]. Furthermore, high p62 expression is positively correlated with the expression of autophagy-related proteins such as LC3A and LC3B, which are associated with extraprostatic invasion. Beclin-1 expression is also associated with extraprostatic invasion, and with lactate dehydrogenase 5—a marker of anaerobic metabolism—and Gleason score in PRAD [104]. Similarly, in breast invasive carcinoma (BRCA), p62 is overexpressed in malignant breast tissue compared with normal breast tissue. Furthermore, treatment with the proteasome inhibitor PSI increases both p62 mRNA and protein levels, but it does not alter the promoter activity of p62 [105]. Moreover, p62 expression is positively associated with human epidermal growth factor receptor 3 (HER3) and HER4, members of the epidermal growth factor receptor (EGFR) family implicated in tumor progression [106]. Triple-negative breast cancer (TNBC) is also associated with the accumulation of p62, which correlates with poor prognosis [107], and the expression of autophagy-related markers, including microtubule-associated protein 1 light chain 3 alpha (LC3A), LC3B, and Beclin-1, is highest in TNBC tumor cells [108]. These observations suggest that p62 accumulation may facilitate metabolic reprogramming and anabolic growth across multiple tumor types.

Dysregulated autophagic flux emerges as another recurrent feature associated with p62 dysregulation. In colon adenocarcinoma, p62 expression is upregulated in colorectal cancer tissues, along with elevated expression of the autophagosome formation marker LC3. Knockdown of p62 expression suppresses autophagy activation and inhibits tumor growth [109]. In head and neck squamous cell carcinoma, accumulation of p62 is frequently observed and is correlated with impaired autophagy. Tumors and cell lines exhibiting high p62 levels demonstrate reduced sensitivity to phosphoinositide 3-kinase/protein kinase B (PI3K/AKT) pathway inhibitors. Analyses of patient samples and Cancer Genome Atlas (TCGA) data have shown that p62 accumulates during progression from dysplasia to carcinoma, accompanied by alterations in ATG7 [110]. In stomach adenocarcinoma, autophagy-related proteins, including LC3, Beclin-1, and p62, have been implicated in hepatic metastasis, vascular invasion, and lymph node metastasis. Moreover, elevated autophagic activity is associated with poor clinical outcomes in patients with gastric cancer [111]. In general, tumorigenesis is suppressed by functional autophagy; however, impaired autophagy results in p62 accumulation, thereby contributing to tumor development [112]. In HCC, p62 accumulation activates NRF2 and mTORC1, underscoring its role in maintaining tumor-initiating cells [101]. The p62–Keap1–NRF2 axis is implicated in both tumorigenesis and tumor suppression. TRIM21, a ubiquitin E3 ligase, promotes HCC progression by inhibiting the p62–Keap1–NRF2 antioxidative pathway [113].

In addition to mechanistic signaling axes, p62 expression also exhibits diverse clinicopathologic correlations across tumor types. In esophageal adenocarcinoma, both LC3B and p62 are expressed, indicating functional autophagic activity. High p62 expression is associated with a more favorable prognosis, whereas tumors with low LC3B and low p62 expression display a more aggressive phenotype and poorer survival outcomes [114]. In pancreatic adenocarcinoma, clinicopathologic analyses have shown that p62 and ubiquitin levels are highly expressed in pancreatic carcinomas, and elevated ubiquitin expression is associated with lymph node metastasis in patients with this cancer, but it does not significantly affect overall survival [115]. In epithelial ovarian cancer, p62, Beclin-1, and excision repair cross-complementation group 1 are overexpressed compared with benign tissues, and BRCA1, Beclin-1, and p62 serve as biomarkers for predicting platinum resistance and prognosis [116], suggesting that p62 expression frequently correlates with clinicopathologic progression and therapeutic response.

Importantly, p62 does not exhibit consistent effects across all malignancies. In uterine corpus endometrial carcinoma (UCEC), suppression of p62 expression is associated with reduced resistance to oxidative stress and decreased invasiveness. Furthermore, in an orthotopic mouse model of endometrial carcinomas (ECs), p62 inhibition attenuates in vivo tumor growth. Collectively, elevated p62 expression may serve as a potential biomarker for ECs [117]. In contrast, in skin cutaneous malignant melanoma, p62 expression is elevated in early lesions but reduced in advanced stages, and low p62 expression is associated with an increased risk of metastasis. Collectively, p62 may serve as a potential prognostic marker in American Joint Committee on Cancer stage II melanoma [118]. Chronic human pancreatitis is associated with the accumulation of p62 and the downregulation of the inhibitor of NF-κB kinase subunit alpha [119], and p62 overexpression enhances bone metastasis and stromal proliferation in bone tumors [120,121].

Recently, Yang et al. demonstrated that p62 is overexpressed in most cancers compared with normal tissues, based on analyses of TCGA datasets and the Gene Expression Profiling Interactive Analysis platform, which provides an algorithm for analyzing 23 types of cancer [14]. In contrast, p62 expression is downregulated in UCEC, pheochromocytoma and paraganglioma, PRAD, and bladder urothelial carcinoma [14].

Collectively, these findings indicate that p62 does not exhibit a consistent biological function across malignancies. Rather, its role is shaped by tumor type, disease stage, autophagic status, inflammatory signaling context, and metabolic demands. Accordingly, p62 should be considered a context-dependent regulator that may function either as an oncogenic driver or as a tumor suppressor depending on the molecular and cellular environment.

**Table 1:** Expression and functional roles of p62 across different cancer types.

Cancer Type	Expression of p62	Associated Pathways/Interacting Factors	References
Breast invasive carcinoma	Overexpressed in malignant tissue	Association with HER3/HER4 (EGFR family)	[105,106,107,108]
Colon adenocarcinoma	Upregulated in cancer tissue	Correlation with ubiquitin and LC3	[109,115]
Liver hepatocellular carcinoma	Highly accumulated	p62 induces activation of NRF2, c-Myc and mTORC1. Phosphorylation of p62 at Ser349	[57,93,94,95,96,101]
Clear cell renal cell carcinoma (ccRCC)	Overexpressed due to chromosome 5q gain	Association with chromosome 5q	[99]
Lung adenocarcinoma/Lung squamous cell carcinoma	Highly accumulated	Correlation with LC3. Association with NRF2 pathway	[97,98]
Pancreatic adenocarcinoma	Upregulated in pancreatic carcinoma	Association with highly accumulated ubiquitin	[115]
Prostate adenocarcinoma	Elevated cytosolic expression in prostatic adenocarcinoma	Association with LC3A and LC3B. Beclin-1 expression was associated with LDH5	[103,104]
Stomach adenocarcinoma	Elavated	Association with LC3 and Beclin-1	[111]
Thyroid cancer	Overexpressed in papillary thyroid carcinoma	Association with AMPK/AKT/mTOR pathway	[102]
Ovarian cancer	Overexpressed in epithelial ovarian cancer	Association with overexpression of Beclin-1, and ERCC1	[116]
Oral cancer	Overexpressed in oral carcinoma	p62 knockdown is associated with accumulation of ROS and reduction in GSH	[100]
Uterine corpus endometrial carcinoma (UCEC)	High expression in tumors	Inhibition of p62 is associated with reduced resistance to oxidative stress and invasiveness	[117]
Skin cutaneous malignant melanoma	Elevated in early stage, reduced in advanced stage	-	[118]
Head and neck squamous cell carcinoma	Frequently accumulated in head and neck squamous carcinoma	Association with PI3K/ATK pathway and ATG7	[110]
Esophageal adenocarcinoma	Expressed with LC3B	Correlation with LC3	[114]

### 4.1. p62 as a Metabolic Regulator in Cancer Therapy

The selective autophagy receptor p62 has long been recognized as a signaling hub. According to a recent study by Xiaochuan Zhang et al., accumulating evidence highlights its critical involvement in cancer cell metabolism, thereby identifying p62 as a promising therapeutic target. Specifically, p62 is associated with glucose, glutamine, and fatty acid metabolism in tumor cells, and with several key signaling pathways [122].

In glucose metabolism, p62 regulates metabolic activity in cancer cells at multiple levels. First, p62 promotes glucose transporter 1 (GLUT1) expression and the mitochondrial localization of hexokinase 2 (HK2), thereby enhancing glucose uptake and glycolysis [123,124]. Clinical HCC samples also demonstrate a positive correlation between HK2 and p62 expression [125]. Moreover, p62 increases the expression of glycolytic enzymes, including GLUT, pyruvate dehydrogenase kinase 1 (PDK1), pyruvate kinase M2 (PKM2), and lactate dehydrogenase A (LDHA), by regulating hypoxia-inducible factor 1-alpha (HIF1α) activity. p62 also modulates the transcriptional activity of HIF1α through the mTORC1 and NF-κB signaling pathways [126]. NF-κB is further associated with p62, thereby promoting glycolysis by upregulating GLUT3 expression [127,128]. In hepatitis B virus (HBV)-infected hepatocellular carcinoma, p62 activates the Keap1–NRF2 axis, which upregulates glucose-6-phosphate dehydrogenase (G6PD) expression and the hexosamine biosynthetic pathway, thereby increasing glutathione synthesis and promoting tumor development [94,129].

In glutamine metabolism, p62 regulates both glutamine utilization and redox homeostasis in tumor cells [122]. It also enhances glutamine uptake via solute carrier family 1 member 5 (SLC1A5) and promotes glutathione biosynthesis through glutamate–cysteine ligase, NAD(P)H quinone oxidoreductase 1, and sulfiredoxin-1 (SRXN1) in tumor cells, whereas p62 deficiency reduces glutamine utilization and nucleotide synthesis [130]. In papillary thyroid carcinoma, p62 knockdown suppresses cell proliferation by inhibiting the AKT/AMPK/mTOR signaling pathway [102], but its phosphorylation at Ser349 directs glutamine flux toward glutathione synthesis, thereby enhancing antioxidant capacity, chemoresistance, and tumor progression [131].

In fatty acid metabolism, p62 plays a multifaceted regulatory role in cancer cells [122]. Fatty acid synthesis requires nicotinamide adenine dinucleotide phosphate, which supports anabolic growth and protects cells against oxidative stress [132]. The p62–ULK1–Keap1–NRF2 axis contributes to ROS elimination [88], while HIF1α enhances fatty acid uptake and lipid storage, thereby promoting cell survival [133]. In addition, p62-mediated autophagy degrades lipid droplets into fatty acids to meet the energy demands of tumor cells. p62 deficiency reduces adipocyte metabolic activity but increases nutrient availability for prostate tumors, further implicating p62 in tumor progression [134].

In the tumor microenvironment, tumor-derived lactate secreted through monocarboxylate transporter 1 (MCT1) acidifies the local environment, promoting adipocyte lipolysis and providing lipids that fuel tumor growth [135]. Elevated lactate levels resulting from p62-mediated glycolysis facilitate immune evasion by polarizing macrophages toward a tumor-promoting M2 phenotype [136]. Furthermore, increased lactate concentrations also influence immune cells such as T lymphocytes and B lymphocytes [137].

### 4.2. Targeting p62 for Cancer Therapy

The multifaceted roles of p62 in cancer metabolism have recently positioned it as a promising therapeutic target [122]. Moreover, enhanced autophagic activity in cancer cells plays a critical role in their survival, making the selective autophagy receptor p62 a key molecular target in cancer therapy [138]. Based on current evidence, most p62-targeting compounds remain in the preclinical stage, with several small-molecule inhibitors under active investigation. According to a recent study by Tetsuya Saito et al., K67 is a small-molecule compound that inhibits the interaction between phosphorylated p62 at Ser349 and Keap1, thereby promoting the degradation of NRF2 through the E3 ubiquitin ligase activity of Keap1 in liver cancer cells and demonstrating that K67 suppressed the proliferation of HCC cells and reduced their tolerance to anticancer agents [94]. Furthermore, in a study by Keiko Tsuganezawa et al., a fluorescence correlation spectroscopy-based competitive binding assay was employed to identify inhibitors of the LC3–p62 interaction, leading to the identification of two compounds with half-maximal inhibitory concentration (IC_50_) values of 0.9 µM and 2.0 µM, respectively [139]. These findings suggest that abnormal autophagic function may be involved. In addition, several compounds targeting the ZZ domain of p62—which participates in the NF-κB signaling pathway—have been investigated, including P62XIE3, XRK3F2, and XIELP1-17b, which specifically target the ZZ domain of p62, exhibiting IC_50_ values of 6.19 µM, 4.35 µM, and 0.84 µM, respectively [122,140]. Furthermore, in a study by Ori Kalid et al., PTX80 was found to interact with p62, leading to a reduction in soluble p62 levels and the aggregation of insoluble p62. This interaction disrupts the colocalization of polyubiquitinated proteins with p62, resulting in the accumulation of abnormal proteins and impaired proteolytic clearance. The resulting proteotoxic stress activates cellular stress responses, including the unfolded protein response, ultimately inducing apoptosis [141].

## 5. Perspective

Although this review focuses on the role of the selective receptor p62 in cancer, recent studies have demonstrated that dysregulated expression of p62 is associated with neurodegenerative diseases, including Alzheimer’s disease (AD), Parkinson’s disease (PD), Huntington’s disease (HD), frontotemporal lobar degeneration (FTLD), and amyotrophic lateral sclerosis (ALS), and other pathological conditions such as age-related macular degeneration (AMD) and diabetes. A hallmark of neurodegenerative diseases is the abnormal accumulation of misfolded proteins in the brain, leading to cellular dysfunction and neuronal death. Moreover, oxidative stress and mitochondrial dysfunction are common pathological features of these disorders [142]. Importantly, p62 has been implicated in the pathogenesis of multiple neurodegenerative diseases, where evidence from various studies indicates that abnormal expression of p62 affects multiple aspects of cellular homeostasis. In these neurodegenerative diseases, aberrant expression of p62 has been associated with impaired clearance of aggregated proteins, dysregulated kinase signaling, and defective mitophagy. Importantly, the functional impacts of p62 dysregulation are not identical. In some diseases, p62 deficiency exacerbates proteotoxic stress and neurodegeneration by impairing protein quality control, whereas in others, its accumulation appears to reflect inhibition of autophagic flux. Collectively, findings from neurodegenerative diseases provide a conceptual basis for the development of p62-dependent autophagy-targeted therapeutic strategies in cancer.

In addition, dynamic changes in p62 levels in cancer are closely associated with autophagic activity. In accordance with the established guidelines for the use and interpretation of assays for monitoring autophagy [143], p62 levels are regulated not only by autophagic degradation but also by transcriptional activity, the ubiquitin–proteasome system, and aggregation. Likewise, LC3-II levels are also regulated not only by autophagosome formation but also by impaired autolysosome activity. Taken together, dynamic changes in p62 and LC3-II levels should be interpreted within a broader context when evaluating autophagic activity in cancer. For example, autophagic flux assays incorporating time-resolved and temporal analyses can more accurately monitor dynamic changes in p62 levels, and such approaches may ultimately provide the development of novel therapeutic strategies targeting autophagy in cancer.

Nevertheless, several conceptual questions remain unresolved. It is still unclear whether p62 accumulation serves as a primary oncogenic driver or rather a secondary consequence of impaired autophagic flux in tumorigenesis. Moreover, although autophagy deficiency frequently correlates with elevated p62 levels, a direct causal relationship between p62 accumulation and cancer initiation has not been fully established. These uncertainties highlight the importance of context-dependent interpretation of p62 dynamics in cancer biology.

## 6. Conclusions

The significance of autophagy in the pathogenesis of various diseases continues to grow, underscoring the increasing importance of research in this field. Among the core components of the autophagic machinery, the selective autophagy receptor p62 plays a pivotal role, and its dysregulation is closely associated with cancer development. In this review, we first provided a comprehensive overview of the three major types of autophagy—macroautophagy, microautophagy, and CMA—and focused on the molecular mechanisms underlying macroautophagy. Subsequently, we discussed the role of p62 as a selective autophagy receptor, including its interactions with various binding partners, the associated signaling pathways, and its diverse cellular functions, and also emphasized that PTMs regulate the phase separation and functional activity of p62. Finally, we highlighted that aberrant expression of p62 is associated with the progression of multiple cancer types. Although considerable progress has been made in understanding p62 biology, several critical questions remain unresolved. For instance, if a deubiquitinating enzyme (DUB) that removes ubiquitin chains binds to p62 and cleaves the polyubiquitin chain from ubiquitinated cargo recognized by p62, the impact of this interaction on autophagic activity may vary depending on the timing and stage at which the DUB acts during the autophagy process. Such temporal regulation of p62 by DUBs could differentially influence cargo recognition, autophagosome formation, or lysosomal degradation. In addition, the appropriate balance between DUBs and E3 ubiquitin ligases is likely to be crucial for maintaining proper p62 levels and functional activity. Dysregulation of this ubiquitination–deubiquitination equilibrium may significantly affect p62 stability, autophagic flux dynamics, and downstream oncogenic signaling in cancer. Future studies should aim to address these questions and identify novel targets, such as DUBs that regulate p62 in a stage-dependent manner, which may provide new therapeutic strategies for cancer treatment. In conclusion, the multifunctional roles of p62 in autophagy and its association with multiple signaling pathways in cancer highlight its potential as a promising therapeutic target in cancer.

## Data Availability

No new data were created or analyzed in this study. Data sharing is not applicable to this article.
